# Collagen IV biosynthesis: Intracellular choreography of post-translational modifications

**DOI:** 10.1016/j.matbio.2025.07.002

**Published:** 2025-07-04

**Authors:** Yoshihiro Ishikawa, Rachel Lennon, Federico Forneris, Johanna Myllyharju, Antti M. Salo

**Affiliations:** a Department of Ophthalmology, University of California San Francisco, School of Medicine, CA, USA; b Manchester Cell-Matrix Centre, Faculty of Biology, Medicine and Health, University of Manchester, UK; c The Armenise-Harvard Laboratory of Structural Biology, Department of Biology and Biotechnology, University of Pavia, Italy; d Fondazione IRCCS Policlinico San Matteo, Pavia, Italy; e Biocenter Oulu, University of Oulu, Finland; f Faculty of Biochemistry and Molecular Medicine, University of Oulu, Finland

**Keywords:** Collagen IV, Type IV collagen, Biosynthesis, Post-translational, Modification, Hydroxylation, Glycosylation, Hydroxylysine, Hydroxyproline, Galactosylhydroxylysine, Glucosylgalactosylhydroxylysine, Endoplasmic reticulum

## Abstract

Collagen IV, an essential and evolutionarily conserved component of basement membranes, is one of the most extensively post-translationally modified proteins. Despite substantial research on fibrillar collagen biosynthesis, our understanding of collagen IV biosynthesis, including its post-translational modifications (PTMs), remains limited. Most PTMs occur intracellularly, primarily within the endoplasmic reticulum (ER). In this review, we examine the molecular ensemble that orchestrates collagen IV biosynthesis in the ER, highlighting the complex interplay between prolyl and lysyl hydroxylases, glycosyltransferases, and molecular chaperones. Furthermore, we discuss how defects in collagen IV and its PTMs contribute to various human pathologies, including Gould and Alport syndromes, fibrosis, and cancer. Understanding collagen IV PTMs is crucial for elucidating the molecular basis of these diseases and improving targeted treatments. By reviewing our knowledge of collagen IV biosynthesis, we illustrate how this evolutionarily conserved yet highly specialized molecular biosynthesis ensemble supports the diverse functions of collagen IV in health and disease.

## Introduction

Collagen IV, the ancestral member in the collagen superfamily, is one of the major components of the basement membrane (BM), a specialized sheet-like extracellular matrix (ECM) structure that is essential for tissues [1–3]. BMs provide structural support to tissues, create a border between cells and tissue compartments, selectively filter molecules, and serve as a cellular signaling platform in the body. Biosynthesis of collagen IV molecules occurs in the endoplasmic reticulum (ER), followed by their secretion and assembly into a supramolecular network-like structure in the extracellular space. The biosynthesis is generally presumed to be well-characterized and understood. However, collagen biosynthesis research and associated literature has extensively focused on fibrillar collagens, especially collagen I. The prevailing assumption that this literature is broadly applicable to non-fibrillar collagens, including collagen IV, oversimplifies the reality. Collagen biosynthesis is likely more specialized and tailored to each collagen type than previously anticipated, a distinction that is even more pronounced for collagen IV. Unlike protease-resistant fibrillar collagens, collagen IV is sensitive to proteolysis, creating a substantial technical barrier for biochemical studies. This barrier has perpetuated an important and persistent gap in our understanding of collagen IV biosynthesis relative to collagen I. Here, we aim to highlight this by raising up outstanding issues in collagen IV synthesis, which are likely to be significant also for understanding collagen biology in general.

The clinical relevance of collagen IV is underscored by the fact that variants in collagen IV genes (*COL4A1* – *COL4A6*) cause the multisystem disorders Gould syndrome and Alport syndrome [4–10]. There is also a common framework in collagen-related diseases: pathogenic outcomes in connective tissues arise not only from defects in collagens themselves but also from pathogenic variants in genes encoding components of the collagen biosynthetic machinery, including the intracellular “molecular ensemble” of ER-resident proteins such as collagen modifying enzymes and molecular chaperones [11]. Thus, understanding collagen biosynthesis is essential for understanding the underlying molecular pathology and identifying novel therapeutic targets. Indeed, variants in the collagen modifying enzyme cause phenotypes that overlap with Gould syndrome, potentially Alport syndrome, as well as other human connective tissue diseases, and their aberrant expression is implicated in fibrosis and cancer.

This review addresses many critical aspects of collagen IV biosynthesis with a particular focus on the intracellular modification steps. First, collagen IV contains evolutionary footprints that indicate both the most changed and most protected biosynthetic machinery over time. Second, collagen IV undergoes more extensive post-translational modifications (PTMs) compared to collagen I, though the precise rules governing these modifications remain undetermined. Third, the lack of focus on collagen IV biosynthesis hinders our understanding of its intricate role in health and disease. Consequently, disruptions in its biosynthesis can have as profound clinical implications as are described *e.g*. in those types of the fibrillar collagen disorders osteogenesis imperfecta (OI) and Ehlers-Danlos syndrome (EDS) that are caused by variants in the collagen biosynthetic machinery [12,13]. By systematically appraising our understanding of collagen IV biosynthesis, we aim to open new horizons for understanding the overall concept of collagen biosynthesis, its modifications, and its properties. This approach will not only clarify the unique biochemical pathways and roles of collagen IV within the BM but also enhance our broader understanding of collagen function and pathology across different tissue types. Such insights are imperative for advancing our knowledge of connective tissue disorders and developing novel therapeutic strategies.

## The life of collagen IV

**Fig. 1:**
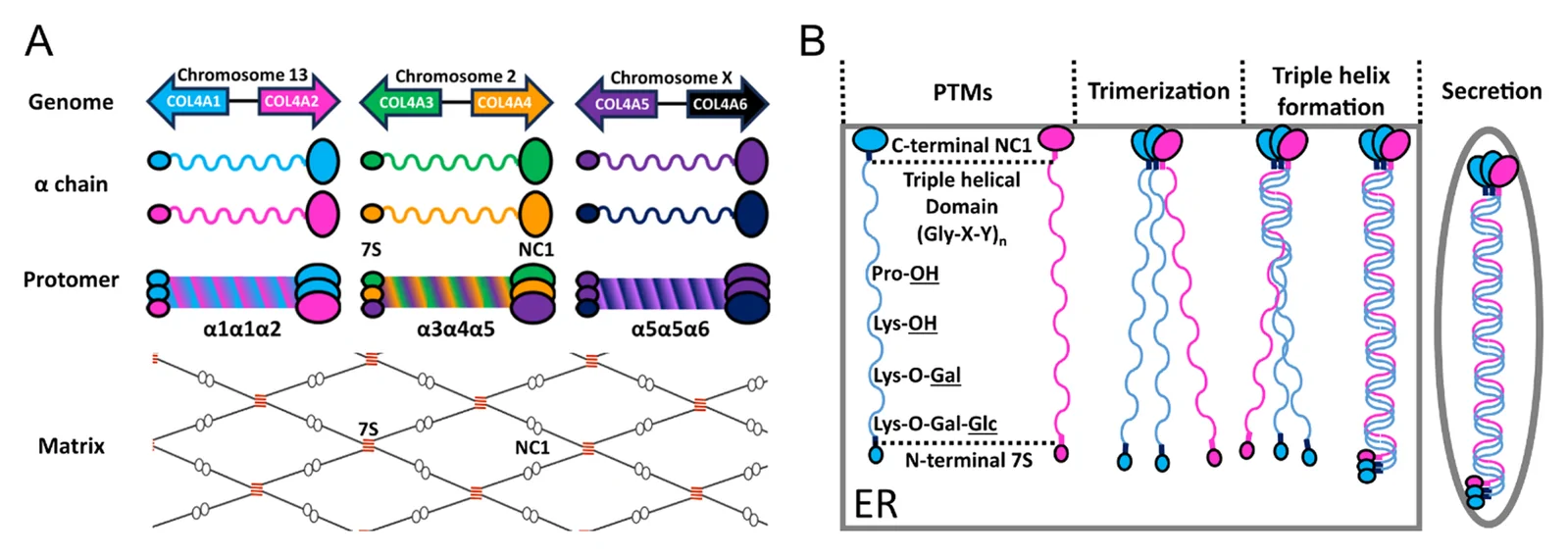
Life of Collagen IV. (A) From Gene to Network: The human genome encodes six collagen IV genes (*COL4A1*-*A6*), located in pairs on chromosomes 13, 2, and X (Genome). Each α chain consists of a collagenous (Gly-X-Y) domain flanked by N-terminal (7S) and C-terminal (NC1) domains (α chain). These α chains assemble into three collagen IV trimers: α1α1α2(IV), α3α4α5(IV), and α5α5α6(IV) (Protomer). These protomers form a sheet-like network through NC1 hexamers and 7S dodecamers in the extracellular space (Matrix). (B) Biosynthesis: Collagen IV biosynthesis is a multi-step process. Newly synthesized collagen polypeptides undergo post-translational modifications (PTMs) including hydroxylation of proline (Pro-OH) and lysine (Lys-OH). Hyl is subsequently glycosylated (Lys-O-Gal, Lys-O-Gal-Glc). Three α chains trimerize and form a triple helix, initiating at the NC1 domain and propagating towards the 7S domain. Properly folded collagen molecules are then secreted into the extracellular space.

Before delving into the details of collagen IV biosynthesis and its molecular ensemble, we briefly highlight the “life” of collagen IV. The human genome encodes six collagen IV genes, *COL4A1*–*A6*, arranged in pair s on three chromosomes: *COL4A1*/*COL4A2, COL4A3*/*COL4A4*, and *COL4A5*/*COL4A6* located on chromosomes 13, 2, and X, respectively (Fig. 1A – Genome). This organization suggests that each pair of collagen IV genes share regulatory control through a common promoter and potentially other regulatory sequences. In contrast, the *COL1A1* and *COL1A2* genes, which encode fibrillar collagen I, reside on different chromosomes (17 and 7, respectively) [14]. The translated nascent collagen IV polypeptide, known as an alpha (α) chain, is consists of three major domains: a central Gly-X-Y collagenous region flanked by amino (N) and carboxyl (C)-terminal domains, termed 7S and NC1 (non--collagenous 1) domains, respectively (Fig. 1A – α chain) [15,16]. Despite the existence of six α chains, only three combinations predominate in nature: α1α1α2(IV), α3α4α5(IV), and α5α5α6(IV), collectively referred to as protomers (Fig. 1A – Protomer) [17,18]. Collagen α1α1α2(IV) is approximately 400 nm in length and longer than fibrillar collagens [19, 20]. These protomers are assembled in the ER and subsequently secreted via the Golgi, a process that we discuss in detail in the following sections. In the extracellular space, these protomers form a specialized sheet-like network through two major macromolecular complexes: NC1 hexamers and 7S dodecamers are formed by head-to-head dimeric protomers and anti-parallel tetrameric protomers, respectively (Fig. 1A – Matrix). This network serves as a critical scaffold, interacting with other ECM proteins (*e.g*. nidogen, SPARC), binding cell surface receptors (*e.g*. integrins, DDR1) and capturing signaling molecules (*e.g*. TGFβ, BMP) [21–27]. As a hub of biological activity, collagen IV plays a critical role in the BM. Matrix metalloproteinases (MMP) 2 and 9 are involved in the remodeling and turnover of this network [28–30], however, the precise turn-over rate of collagen IV across different tissues and across development and aging remains unclear. For more in-depth discussions on other aspects of collagen IV biology beyond its biosynthesis, we refer readers to the many excellent review articles available on this topic [3,9,16,31–33].

## How collagen IV is made: A blueprint of biosynthesis

The overall process of collagen biosynthesis in the ER is a highly complex but orchestrated process carried out by a molecular ensemble of proteins (Fig. 1B). The ensemble involves around 20 ER-resident proteins, which play direct roles in ensuring efficiency and quality [11,34–37]. Newly synthesized collagen polypeptides undergo extensive yet precise PTMs within the ER lumen, which are essential pre-requisites for triple helix formation and can also have direct functional effects. Unlike multidomain proteins that fold co-translationally, the formation of the collagen triple helix requires nucleation at the C-terminal region. This registration process subsequently initiates and propagates in a manner akin to braiding a rope, extending toward the N-terminus [37–41]. Only molecules with a properly formed triple-helical structure, verified through quality control mechanisms under the guidance of molecular chaperones, are permitted to proceed to the extracellular space.

**Fig. 2:**
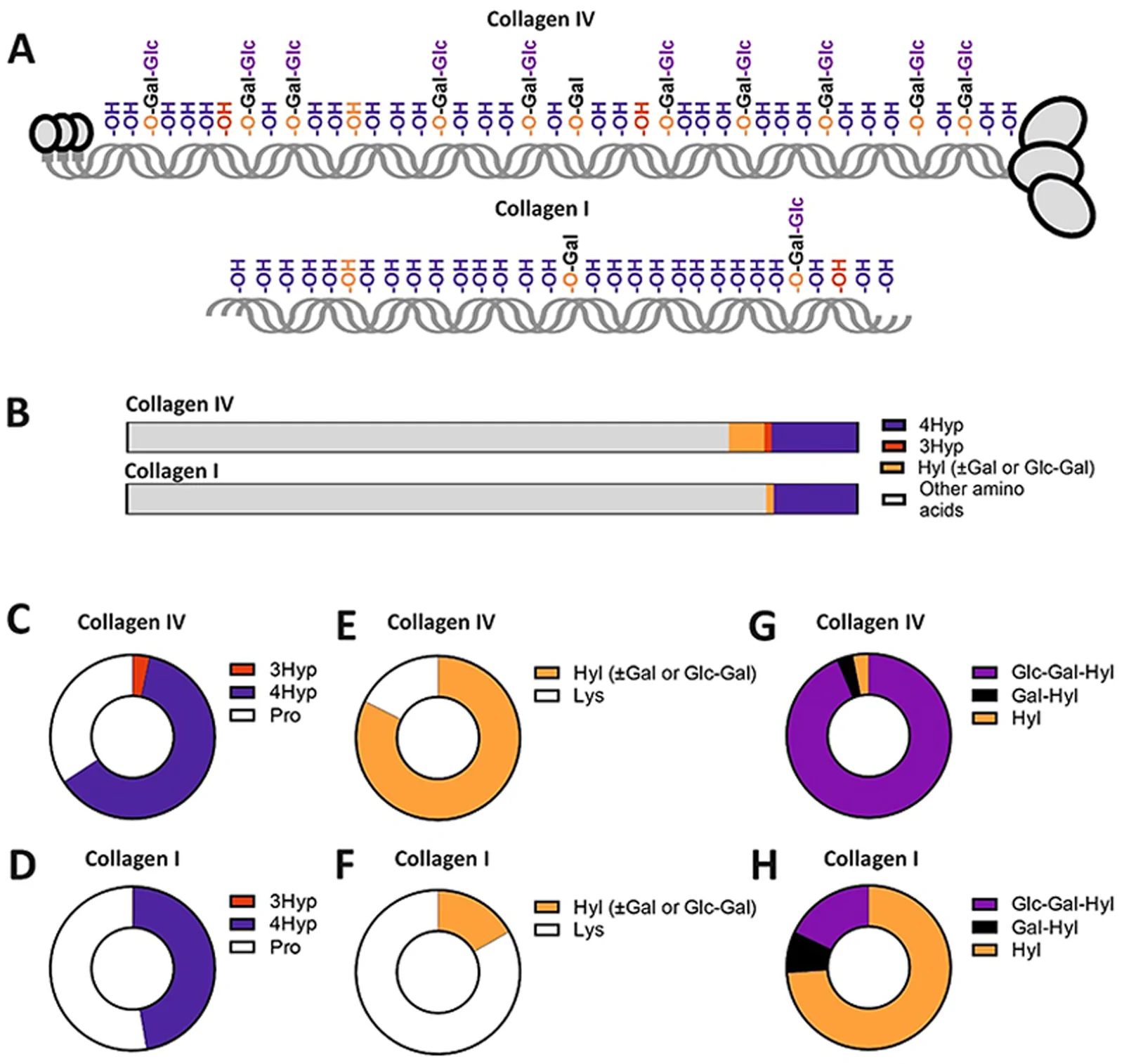
Collagen IV is heavily decorated with post-translational modifications. (A) Cartoon representation of collagen IV and collagen I to highlight especially the more abundant Hyl, glycosylated Hyl and 3Hyp in collagen IV. 3Hyp marked with red, Hyl with orange and 4Hyp with blue. Gal-Hyl in black and Glc-Gal-Hyl with purple. (B) The quantity of proline and lysine PTMs in collagen IV and collagen IV per 1000 amino acids. Values are from ref [42]. C and D) Comparison of relative proline modification levels in collagen IV and I, respectively. E and F) Comparison of relative Lys modification levels in collagen IV and I, respectively. G and H) Comparison of relative Hyl glycosylation levels in collagen IV and I, respectively. The values in C and E are from ref [22], values in D, F and H are from ref [43], values in G are from ref [44]. Hyl values in B, E and F include the Hyl itself and its putative glycosylated derivatives Gal-Hyl and Glc-Gal-Hyl.

While many aspects of collagen biosynthesis are conserved across different collagen types, PTMs exhibit remarkable diversity, providing a distinct molecular signature for each collagen. Collagen α1α1α2(IV) undergoes extensive PTMs, making it one of the most heavily modified collagens (Fig. 2). This suggests that a highly specific PTM machinery is required in collagen IV biosynthesis. Before exploring the detailed roles of PTMs in later sections, we highlight other unique aspects of collagen IV biosynthesis compared to other collagen types.

## Collagen IV formation, quality control and trafficking

### NC1 domain assembly

Despite six genes (*COL4A1*-*COL4A6*) encoding collagen IV chains, only three combinations predominate in nature: α1α1α2(IV), α3α4α5 (IV), and α5α5α6(IV). This specific selection of chains for each trimer is crucial for the proper function of collagen IV networks, thereby establishing distinct networks with unique properties and functions in different tissues. Collagen IV triple helix formation begins with the assembly of C-terminus NC1 domain trimers [45]. In the low chloride concentration of the ER, NC1 trimers are unstable and they do not dimerize. Formation of a stable NC1 trimer requires a triple-helical portion extending from the NC1 complex [46]. This suggests that nucleation and propagation of the collagen IV triple helix are initiated synergistically by two factors: the interaction of three NC1 domains with an appropriate stoichiometry and the winding of the C-terminal collagenous region. Once secreted into the extracellular space, where the chloride concentration is higher, two NC1 trimers dimerize into a stable hexamer, which is an integral step in the collagen IV network formation [46,47]. In fibrillar collagens, the C-terminal propeptide (C-pro) domains serve an analogous role as the NC1 domains [48] However, C-pro trimer assembly relies on three factors: calcium ions, interchain disulfide bonds, and N-glycosylation [49–54]. This suggests that the collagen IV NC1 assembly is more self-sufficient.

### Triple helix propagation

Once the collagen IV chains assemble with the correct stoichiometry, the triple helix zips up from the C-terminus to the N-terminus, guided by the characteristic sequence of collagen. The collagenous sequence consists of Gly-X-Y repeats, where X and Y are variable amino acids, frequently proline and 4-hydroxyproline, respectively. Collagen α1α1α2 (IV) spans ~400 nm and includes regions with interruptions in the Gly-X-Y motif contributing to the flexibility of collagen IV [19,20,55]. Importantly, during helix formation in all collagens, proline residues must isomerize from the *cis* to the *trans* state, since proline peptide bonds in the triple helix adopt the *trans* configuration. This rate-limiting step is facilitated by ER-resident peptidyl-prolyl *cis*/*trans* isomerases (PPIases) [41,56–58]. In collagen I, delayed triple helix formation can lead to excessive hydroxylation and glycosylation due to prolonged exposure of the nascent chains to collagen modifying enzymes [59–62]. This affects fibril packing and is frequently observed in pathologies such as OI [63]. This highlights the importance of PPIases in regulating triple helix formation and their involvement in collagen I related diseases [61,64–67]. In contrast, *in vivo* biosynthesis of collagen α1α1α2(IV) is significantly slower than that of fibrillar collagens (7–8 h *versus* 1–2 h) [44]. Although this slower rate correlates with its extensive PTMs, its refolding rate *in vitro* is unexpectedly similar to that of fibrillar collagens [41,45,68]. Interestingly, bacterial PPIases can also accelerate the refolding of collagen α1α1α2(IV) *in vitro* [45]. These findings hint at a sophisticated interplay between PTMs and folding rates in the presence of PPIases in the ER.

### Role of prolyl isomerases and molecular chaperones in the endoplasmic reticulum

Seven PPIases reside in the ER, divided into two families: the FK506-binding protein (FKBP) and the cyclophilin (Cyp) families. Six of them belong to the FKBP family, with protein names reflecting their molecular weights. The gene names differ from the protein names and are shown in parentheses: FKBP13 (*FKBP2*), FKBP19 (*FKBP11*), FKBP22 (*FKBP14*), FKBP23 (*FKBP7*), FKBP60 (*FKBP9*), and FKBP65 (*FKBP10*). Each FKBP protein possesses at least one FKBP domain that catalyzes prolyl isomerization and binds the immunosuppressant drug FK506, which is now known as tacrolimus [38,39,69,70]. The seventh PPIase, CYPB (*PPIB*), belongs to the cyclophilin family and represents the most abundant ER PPIase [71]. These PPIases can catalyze prolyl isomerization, although their activity varies depending on the amino acid combinations in the X-Pro bond and the presence or absence of proline hydroxylations [39,72,73]. This suggests that they could, in theory, facilitate collagen triple helix formation in all 28 collagen types. However, this possibility remains largely unexplored due to the difficulty of performing collagen folding assays *in vitro*, even for collagen I [37]. In humans, genetic variants in *FKBP14, FKBP10*, and *PPIB* (encoding FKBP22, FKBP65 and CYPB, respectively) can lead to heritable connective tissue disorders. Pathogenic variants of *FKBP14* cause kyphoscoliotic Ehlers-Danlos syndrome (kEDS) (as does *PLOD1* mutations) [74–76], whereas variants of *PPIB* can cause type IX OI and variants in *FKBP10* can cause type XI OI or Bruck syndrome 1 (BRKS1) (of note, *PLOD2* variants cause phenotypically similar BRKS2) [64,66,77,78]. These disorders pre-dominantly result in defects in fibrillar collagens I, III, and V, suggesting that these three PPIases play key roles in the biosynthesis of these fibrillar collagens. However, the specific roles and/or specificity of PPIases in collagen α1α1α2(IV) biosynthesis remain to be fully elucidated. It is important to note that FKBP22 functions not only as a PPIase but also as a molecular chaperone during biosynthesis of collagen III, but not collagen I, due to preferential binding to triple-helical collagen III [39,79]. Interestingly, FKBP22 also binds to triple-helical collagen α1α1α2(IV) and collagen VI and X similar to collagen III [76,79]. FKBP65 similarly exhibits molecular chaperone activity [80,81]. Moreover, FKBP65 and CYPB form complexes with other collagen-related ER proteins, including prolyl 3-hydroxylase (P3H) and lysyl hydroxylase (LH), as discussed later [81–86]. Collectively, their multi-functionality and interactions with other molecules could hold the key to understanding their roles in collagen biosynthesis beyond their prolyl isomerase activity.

The ER plays a critical role in triaging misfolded proteins from the secretory pathway, a process termed “quality control” [36,87–89]. Heat Shock Protein 47 kDa (HSP47) serves as the primary molecular chaperone in collagen biosynthesis [90,91]. Interestingly, recent studies have revealed that HSP47 participates in various biological processes beyond collagen biosynthesis, including its roles as a regulator of the unfolded protein response transducer [92], gravity sensor [93], and immune cell activation [94]. The absence of HSP47 results in the failure to form BMs, highlighting its indispensable role [95–97]. Apart from HSP47, less is known about other potential chaperones involved in collagen IV biosynthesis. As noted in the PPIase section, triple-helical collagen α1α1α2(IV) is among the collagen types with which FKBP22 interacts [76]. We anticipate that this binding indicates a role of a molecular chaperone, however the specific chaperone functions remain largely unclear. In addition, it is still unclear whether the PPIase activity of FKBP22 is involved in the triple helix formation of collagen α1α1α2(IV). Further investigation is needed to elucidate the function of FKBP22 and identify other potential chaperones involved in collagen IV biosynthesis.

### Trafficking to the extracellular matrix

Intracellular trafficking of large cargo proteins, collagens in particular, remains a subject of intense scientific debate. The controversy stems from a fundamental paradox: conventional COPII vesicles have diameters of only 60–90 nm [98–101], seemingly too small to accommodate the rigid, elongated structure of collagen molecules. The discovery of TANGO1 (Transport and Golgi Organization 1) in 2009 [102] revolutionized our understanding of collagen trafficking. TANGO1 facilitates collagen export at the ER exit site by leveraging an extended intra-ER arm in combination with cytoplasmic domains [103–107]. In this system, HSP47 acts as a molecular bridge between collagens and TANGO1 [108] via the Src-homology 3 (SH3) domain positioned at the tip of the intra-ER arm of TANGO1, effectively sandwiching HSP47 between collagen and TANGO1.

Fluorescent imaging techniques, such as GFP-tagging, have become powerful tools for tracking intracellular secretory pathways. A growing number of studies have employed fluorophore-tagged collagen molecules in both cellular and animal models [109–117]. Despite these technological advances, convincing evidence for a simple collagen trafficking mechanism remains elusive. This complexity is underscored by recent findings on collagen α1α1α2(IV) secretion. One study, using live-cell imaging of GFP-tagged α1(IV) chain [118], suggests that the secretory pathway for collagen α1α1α2(IV) may not follow the TANGO1 model. The flexibility of collagen α1α1α2(IV), due to interruptions in its triple-helical structure, that renders it biophysically distinct from fibrillar collagens, might even allow it to bypass the TANGO1-mediated pathway [20,119]. However, the intracellular accumulation of collagen α1α1α2(IV) in TANGO1 knockout mice introduces a puzzling paradox [120].

When interpreting fluorescence-based studies in collagen biology, a cautious approach is warranted, as verifying the formation of the proper triple helix is essential. While fluorescence imaging is visually appealing, it should ideally be complemented with additional methods to confirm that the observed fluorescent signals represent correctly assembled, trimeric collagen molecules. Other potential pitfalls associated with the use of fluorescence-tagged proteins in the BM have been well documented [121]. In summary, current evidence indicates that while TANGO1 and HSP47 are undoubtedly crucial for collagen secretion, their precise roles, type preferences, and the detailed pathways they mediate remain significant subjects of ongoing investigation.

## Intracellular post-translational modifications

Collagens are unique proteins in that their proline (Pro) and lysine (Lys) residues are abundantly modified by hydroxylation and further by hydroxylysine glycosylation to form 4-hydroxyproline (4Hyp), 3-hydroxyproline (3Hyp), 5-hydroxylysine (Hyl), and glycosylated Hyl derivatives galactosyl-Hyl, and glucosylgalactosyl-Hyl (Glc-Gal-Hyl), respectively. The relative amount of 4Hyp is rather similar between collagen types, whereas the abundance of 3Hyp, Hyl, Gal-Hyl and Glc-Gal-Hyl varies more [42]. Similarly, with exception of 4Hyp, the abundance of these modifications also shows differences within the same collagen type extracted from different tissues. Collagen IV is among the most heavily modified collagens regarding the amount of 3Hyp, Hyl and glycosylated Hyl residues (Fig. 2). This part of the review will concentrate on the generation and roles of these modifications and the responsible enzymes. One of the key regulators of collagen synthesis is ascorbate and it functions via PTM formation. Many of the modifying enzymes (prolyl and lysyl hydroxylases) are 2-oxoglutarate dependent dioxygenases that require ascorbate for sustained function. This requirement should always be considered also in cell and organ culture studies by frequent supplementation of fresh ascorbate, or its more stable forms, into culture medium to ensure proper collagen synthesis.

## Proline post-translational modifications - 4-Hydroxyproline

The molecular function of 4Hyp is to provide physiological thermal stability for the triple helix of collagen molecules, including collagen IV [122,123]. 4Hyp in the Y-position stabilizes the collagen triple helix through a stereoelectronic effect on the proline ring conformation [124]. Furthermore, besides being essential for the triple helix stability, 4-hydroxylation of the Y proline and the resulting ring pucker can further affect an X position proline ring pucker, giving it defined molecular flexibility, which may be of functional significance [125]. The 4Hyp residues are also likely to have other functions besides being essential for the structural stability of the triple helix. For example, integrin binding motifs of collagens often contain 4Hyp and its importance has been demonstrated in binding to collagen I, the effect being mediated by both a direct mechanism and stabilization of the triple helix [126]. Collagen IV has many interactors such as integrins, nidogen, fibronectin, perlecan, HSP47, SPARC, DDR1 and von Hippel-Lindau protein (VHL) [21, 24,25,28,127]. Interestingly, the interaction between VHL and unfolded collagen IV chains has been demonstrated to be specific, direct, and 4Hyp-dependent [128].

Prolyl 4-hydroxylation in collagen takes place in the Y-position Pro residues of X-Y-Gly sequences to form X-4Hyp-Gly from X-Pro-Gly. The sequence of the canonical Gly-X-Y triplet repeat of collagen is written here in the X-Y-Gly order as the minimum substrate requirement for the prolyl 4-hydroxylation reaction is fulfilled by an X-Pro-Gly peptide, but not by a Gly-X-Pro peptide [129]. 4Hyp has been identified in collagens by amino acid analyses, sequencing of cyanogen bromide fragments, and more recently by mass spectrometry analyses [44,130–135]. These studies have shown that 4Hyp is a very abundant modification in collagens, collagen IV being no exception (Fig. 2). 148 and 163 4Hyp sites were identified in mouse and human collagen IV chains with a sequence coverage of > 80 % [136]. This translates into 70 % and 76 % hydroxylation of all Y-position prolines in mouse and human collagen IV chains, respectively, which is in agreement with previous studies on collagen IV [44,130–133], and show that, similarly to collagen I, the overall degree of hydroxylation of the Y-position prolines in collagen IV is high, but not entirely complete.

Formation of the 4Hyp residues in collagens is catalyzed by collagen prolyl 4-hydroxylases (C-P4Hs). They, as well as the P3Hs and LHs, are 2-oxoglutarate dependent dioxygenases that require 2-oxoglutarate, molecular oxygen and Fe^2+^ as co-substrates and co-factors in a reaction, where an oxygen atom derived from the molecular oxygen is transferred to the recipient amino acid [137]. In addition, sustained C-P4H activity requires ascorbate. This is because even in saturating concentrations of a collagen substrate C-P4H catalyzes at a low rate so called uncoupled reactions, *i.e*. reactions where decarboxylation of 2-oxoglutarate is not coupled to hydroxylation of the proline residues. In such cases, ascorbate is required as an alternative oxygen acceptor. A well-known example of the importance of ascorbate for C-P4H function is scurvy, a disease caused by lack of vitamin C [138,139]. C-P4H was first purified from vertebrate tissues in 1970 [140] and cloned in 1989 [141,142]. It is an α_2_β_2_ tetramer with two catalytic α subunits (encoded by *P4HA1, P4HA2* and *P4HA3*) and protein disulfide isomerase (PDI, encoded by *P4HB*) serving as the β subunit. Vertebrate C-P4H tetramers always have two identical α subunits, resulting in three C-P4H iso-enzymes, I, II and III, depending on the α subunit [122,143–146]. Instead, in *C. elegans* the main C-P4H form is a tetramer with two different α subunits (PHY1 and PHY2) and in the absence of the other, they can also form active dimers with the β subunit [147], all being forms that do not exist in vertebrates. The function of the PDI/β subunit is to provide solubility to the otherwise completely insoluble and inactive α subunit and PDI also harbours the C-terminal ER retention motif to keep the C-P4H tetramers in the lumen of the ER [122].

The catalytic α subunit consists of three domains, an N-terminal dimerization domain, a central peptide-substrate-biding (PSB) domain and a C-terminal catalytic domain. The N-terminal dimerization domain forms an antiparallel coiled-coil dimer interphase between the two α subunits [148,149]. The PSB domain binds various proline-rich peptide substrates and inhibitors of C-P4H and has a tetratricopeptide repeat (TPR) structure, which is known to be involved in many protein-protein interactions [150–152]. The PSB domain has an important role in the hydroxylation that occurs in the catalytic domain [150], but how these two domains exactly co-operate is not known. Interestingly, C-P4H-I and II exhibit differences in their catalytic and inhibitory constants for (Pro-Pro-Gly)_n_ substrates and poly-L-proline inhibitors, and similar differences are observed in the binding affinities of these peptides to the corresponding PSB domains [151,153]. For example, poly-L-proline is a potent inhibitor of C-P4H-I and binds effectively to PSB-I, while this is not the case for C-P4H-II and PSB-II [153]. The catalytic domain of C-P4H, characterized by the double-stranded beta helix (DSBH) scaffold [149], contains a conserved -HisXAsp/Glu…His- motif [154] that is brought together into a correct structural conformation for Fe^2+^ coordination by the DSBH core fold. The conserved -HisXAsp/Glu…His-motif and the DSBH fold are hallmarks of the catalytic regions of all members of the large 2-oxoglutarate-dependent dioxygenase family [155] that includes also the collagen P3Hs and LHs (see below).

The molecular architecture of the complete C-P4H tetramer has not yet been successfully obtained experimentally, and the current model of its structure is a combination of crystal structures of the individual α subunit domains and the PDI/β subunit, low resolution small-angle X-ray scattering analyses and computational structure predictions [149]. In this model the PSB and catalytic domains are located in the ends of the rod-like antiparallel dimer created by the dimerization domains of the two α subunits so that the catalytic domains interact with the two PDI/β subunits.

Recent results show that the main C-P4H isoforms I and II exhibit both redundancy and specificity towards hydroxylation of various X-Pro-Gly sites [134,135]. Lack of C-P4H-I results in underhydroxylation of several, but not all sites in collagen I, while lack of C-P4H-II has a more restricted effect, it is specifically needed to hydroxylate sites where the X-position is occupied by an acidic residue, that is, Glu-Pro-Gly and Asp-Pro-Gly sites [134,135]. Besides collagen I, similar effects upon lack of C-P4H-II was demonstrated in collagen IV hydroxylation [135]. The hydroxylation site specificity of the two C-P4H isoenzymes was found to be governed by the active site where the selectivity is largely controlled by the X-position amino acid of the substrate although other sequence context also played a role. Despite these differences in the hydroxylation of specific X-Pro-Gly triples, the two C-P4H isoenzymes do not display overall collagen type specificity as collagen IV (as well as collagen I and many other collagen types) can be efficiently hydroxylated by recombinant C-P4H-I and C-P4H-II *in vitro* [135]. It is worth noting that underhydroxylation of Asp-Pro-Gly sites in C-P4H-II null mice was even more drastic than that of Glu-Pro-Gly sites. Interestingly, Asp-Pro-Gly sites are far more abundant in collagen IV than in collagen I, ranging from 6 sites in the α1(IV) chain to 16 sites in the α4(IV) chain compared to a single site in the α2(I) chain and none in the α1(I) chain. Furthermore, some of the Asp-Pro-Gly sites are located in collagen IV near the sites where the triple helix structure is interrupted. Thus, it is possible that lack of C-P4H-II activity and the resulting underhydroxylation of these sites might affect collagen IV biology in a yet unknown manner.

C-P4H activity has been shown to be crucially important for collagen biosynthesis, including collagen IV synthesis and assembly. Genetic deletion of C-P4H-I, the most abundant isoenzyme, in mouse results in a major loss of collagen IV in BMs, disruption of BMs and early embryonic lethality at embryonic day 10.5, that is, already before the time point when abundant synthesis of fibrillar collagens starts [156]. In contrast, loss of C-P4H-II has only minor consequences on overall collagen synthesis and assembly [157,158] and, for example, the ultrastructure of skin capillary BM was only marginally affected, while no structural abnormalities were found in dermal-epidermal BMs [135]. Furthermore, compound mutant *P4ha1*^+/-^,*P4ha2*^-/-^ mice are also viable with a normal lifespan, but have abnormalities in skeletal development resulting in chrondrodysplasia [157,158]. In line with the *in vivo* mouse studies, cells with reduced total C-P4H activity have been shown to have a reduced amount of collagen IV, as well as laminin, in the culture medium [159].

In conclusion, 4Hyp is crucially important for collagen IV structure and C-P4H-I has a major role in this modification, while C-P4H-II may have a more specialized role. This is further emphasized by a recent study showing that knockdown of *P4HA1* in human kidney podocytes results in reduced secretion of collagen IV, particularly under conditions mimicking the blood flow conditions that the glomerular capillary walls encounter [160].

## Proline post-translational modifications - 3-Hydroxyproline

Prolyl 3-hydroxylation is much less frequent in collagens, including collagen IV, than prolyl 4-hydroxylation [42,136] (Fig. 2) and the biological role of 3Hyp is still poorly understood. The 3Hyp residues have been suggested to have a role in collagen stability [161], fibril formation [63,162], molecular interactions [22,163,164] and to have consequences on collagen quantity [165]. It is possible that different 3Hyp sites have different purposes.

Prolyl 3-hydroxylation occurs in collagens in the X-position Pro of Pro-4Hyp-Gly sites generating 3Hyp-4Hyp-Gly, that is, 3-hydroxylation of the X-position Pro is preceded by 4-hydroxylation of the Y-position Pro [166,167]. Interestingly, 3Hyp content is higher in collagen IV than for example in collagen I [168–171]. Basak and co-workers, for example, identified 7 and 14 3Hyp residues in mouse and human collagen IV chains, respectively [136], while human collagen I chains were originally thought to contain only one 3Hyp site. However, additional sites in type I collagen have since been reported, many of them showing tissue-specific differences in the occupancy of 3Hyp [171,172].

Formation of 3Hyp residues is catalyzed by collagen P3Hs. Vertebrate P3H was first partially purified from chick embryo extract in 1979 [173] and cloned in 2004 [82] upon which the P3H protein family (LEPRE) was found to consist of three isoenzymes (P3H1, P3H2 and P3H3) and two catalytically inactive members SC65 and cartilage-associated protein (CRTAP) [82,171,174]. These proteins are encoded by the *P3H1, P3H2, P3H3, P3H4* and *CRTAP* genes, respectively. Gene names *LEPRE1, LEPREL1, LEPREL2, LEPREL4*, and *CASP*, respectively, are also often used. Interestingly, P3H1 was found to be co-eluted with CRTAP and CYPB. The P3H1, CRTAP and CYPB arrange into a 1:1:1 stoichiometric complex [82] and the complex members generally show stability dependence on each other [175,176]. Cryo-EM structure of the complex shows that the active sites of P3H1 and CYPB form a bifunctional face-to-face reaction center for the hydroxylation and isomerization reactions [177]. Furthermore, several collagen recruitment sites were detected in the complex. Intriguingly, although P3H1 does not have a similar PSB domain as the C-P4H α subunit, two of the collagen binding sites in the P3H1 complex are formed by TPR-motifs of CRTAP [177] in an analogous manner to the TPR motifs of the C-P4H PSB domain. The biological importance of the P3H1:CRTAP: CYPB complex formation and the importance of prolyl 3-hydroxylation of collagen in general is highlighted in osteogenesis imperfecta, where human variants have been identified in all three genes [178]. All three P3H isoenzymes contain a canonical C-terminal ER retention signal [82].

P3H1 immunochemistry showed that P3H1 is localized in tissues that express fibrillar collagens and it was suggested that other P3Hs may be responsible for BM collagen hydroxylation [82]. Indeed, the second isoform P3H2 has been identified to be important in collagen IV hydroxylation [163,179,180]. P3H2 was found to be strongly expressed in BM-rich tissues and recombinant P3H2 hydroxylated more effectively synthetic collagen IV peptides than collagen I peptides [179,181]. The collagen IV – P3H2 connectivity is strongly supported by P3H2 mouse models. P3H2 knockout mice exhibited early embryonic lethality in one study [163], while in another study P3H2 null mice were viable without any gross musculoskeletal phenotype, but had high myopia and structural alterations in many eye tissues [182]. The lethality is thought to involve interaction of 3Hyp-deficient collagen IV of the embryo with the maternal platelet-specific glycoprotein VI (GPVI), which results in maternal platelet aggregation and blood thrombosis and death of the mutant embryos [163]. A key finding in both studies was that loss of P3H2 results in significant loss of 3Hyp in collagen IV, although it can also affect collagen I [163,182]. The P3H2-catalyzed 3Hyp residues in collagen IV have been suggested to affect the interaction landscape of collagen IV by affecting its binding to GPIV and nidogens 1 and 2 [163,182]. Furthermore, kidney podocyte-specific loss of P3H2 in mouse results in a thin BM nephropathy phenotype that progresses into focal segmental glomerulosclerosis with microhematuria and micro-albuminuria [165]. Proteomic analyses showed that the amount of 3Hyp residues in collagen IV chains and the abundance of the collagen IV chains and their interacting proteins were significantly reduced in the glomerular BM of the mutant mice. Currently it is not known whether P3H2 has binding partners and whether it forms a supramolecular complex like P3H1. P3H3 forms a complex with LH1, SC65, and CYPB that is involved in collagen I crosslink formation [43,183]. The latest literature thus implies that forming complexes among the components of the collagen molecular biosynthesis ensemble is likely to be necessary for effective PTM formation in fibrillar collagens. Notably, while P3H1 and P3H3 are known to be involved in these complexes, P3H2 appears to function independently so far. Interestingly, recombinant expression of the human P3H1 and P3H2 alone in insect cells showed that although most of the P3H1 and P3H2 protein was insoluble, both were also present in a soluble form, the solubility of P3H2 being lower. However, despite its lower solubility, only P3H2 had P3H activity indicating that unlike P3H1 it can function as an active soluble enzyme without any binding partners [179]. The substrate of P3H3 is not known and no loss of 3Hyp has been demonstrated for P3H3 deletion. Interestingly, loss of P3H3 in mouse phenocopies the type I collagen cross-link abnormality observed in human kEDS patients caused by LH1 variants and in LH1 null mice with lysine underhydroxylation of collagen I and V [43,183,184]. To our knowledge, the molecular mechanism how lysine hydroxylation is controlled by P3H3 is not known. Interestingly, similar lysine underhydroxylation was observed also in SC65 null mice [183].

In summary, 3Hyp is important for correct collagen IV and BM structure and function and P3H2 is the major 3Hyp-catalyzing enzyme for collagen IV.

## Lysine post-translational modifications – hydroxylation and subsequent glycosylation

PTMs of collagen lysine residues include intracellular hydroxylation and subsequent glycosylation, as well as extracellular cross-linking [11,122,185]. Intracellular and extracellular lysine modifications are uncoupled: both unmodified and hydroxylated/glycosylated lysine residues can undergo cross-linking through the action of lysyl oxidases. The fine balance between lysine and hydroxylysine cross-links is critical for imparting tensile strength and extracellular structural integrity in both fibrillar and non-fibrillar collagens. In fibrillar collagens, defects in the collagen lysine PTM machinery cause connective tissue disorders such as kEDS [186,187] and Bruck syndrome 2 [188,189]. Hydroxylysine glycosylations are essential for the secretion of non-fibrillar collagens IV and VI and the final assembly of BMs [44,190–192] and were found to modulate the collagen IV binding properties of cell surface receptors [193,194]. Collagen IV is heavily decorated with PTMs (Fig. 2). For example, Basak and co-workers identified 39 glycosylation sites from mouse α1(IV) chain with sequence coverage of 81.5 % [136]. This result is in agreement with other studies that show approximately 80 % Hyl and only 20 % Lys of total Lys in collagen IV [22,44]. It is remarkable that of these Hyl residues, over 90 % can be glycosylated, the vast majority being Glc-Gal-Hyl [44].

The path to intracellular collagen lysine PTMs features chemical and enzymatic complexity. Firstly, collagen lysine residues can be subject to sequential reactions resulting in different PTM patterns. Firstly, the lysine side chain can be hydroxylated (*i.e*. LH activity), resulting in the generation of Hyl residues. In turn, the hydroxyl moiety of Hyl can become the site for O-linked galactosylation (*i.e*. Gal-T activity), yielding β-(1,O)-galactosyl-5-hydroxylysine (Gal-Hyl). The glycosylation pattern is further extended through glucosylation of Gal-Hyl (*i.e*. Glc-T activity), ultimately producing α-(1,2)-Glc-β-(1,O)-Gal-5-hydroxylysine (Glc-Gal-Hyl) [122,185,195]. The three chemical reactions responsible for Lys to Glc-Gal-Hyl conversion are catalyzed through the alternate action of two enzyme families. Multifunctional lysyl hydroxylase (LH/PLOD, hereafter named LH) enzymes, encoded by the *PLOD* genes, other than performing the eponymous 5-hydroxylation reaction, can carry out Gal-Hyl glucosylation after specific Hyl galactosyltransferases belonging to the GLT25D/COLGALT family, (hereafter named GLT25D), encoded by *COLGALT* genes, have performed their reaction on Hyl products [196].

LH was first purified from chick embryos and human placental tissues in the 1970s - early 1980s [122,197] followed by cloning and identification of the human LH enzyme family that incorporates three isoforms (*i.e*. LH1, LH2, and LH3 encoded by the *PLOD1, PLOD2*, and *PLOD3* genes, respectively) sharing around 70 % sequence identity [198–201]. Regarding the LH reaction, currently available data do not highlight marked specificity for different collagen lysine residues by distinct isoenzymes except for LH2, which shows clear preference for the telopeptide regions of fibrillar collagens that take part in cross-linking, while LH1 and LH3 instead operate on lysine residues located in triple-helical regions [202–204]. Cellular studies indicated that LH3 hydroxylates more selectively collagen IV lysine residues [44], whereas LH1 extensively contribute to collagen I lysine hydroxylation [43,186,205]. *In vitro* and *in vivo* data about the ability of these enzymes to carry out Gal-Hyl glucosylation do not provide additional indications regarding preference for specific sequences surrounding the Gal-Hyl substrate residues, but highlight much higher enzymatic activity in LH3 compared to the other two isoforms, possibly suggesting that LH3 could be responsible for most of the Glc-T activity *in vivo* [44,206–209]. Another surprising feature of LHs is the absence of ER retention sequences at their C-terminus, suggestive of dedicated mechanisms to ensure their localization within the correct sub-cellular compartment during collagen biosynthesis [210]. Unexpectedly, however, LH2 and LH3 have been shown to be present also in the extracellular space as active enzymes [208,211–214].

The GLT25D enzymes, responsible for Gal-T activity, were discovered decades after the initial identification of LH [196]. Prior to GLT25D discovery and characterization, hypotheses were made regarding the possible ability of the LHs to perform the entire Lys to Glc-Gal-Hyl conversion [215], but the observed extremely low Gal-T activity levels *in vitro* already suggested the need for dedicated additional enzyme systems for collagen lysine galactosylation *in vivo* [206]. Similar to LH, also GLT25D displays multiple isoforms in humans. In this case, evidence has been collected at the tissue level, highlighting constitutive expression and ubiquitous presence of the active GLT25D1 isoform and more specific localization of the GLT25D2 isoform at low levels in the nervous system and in cartilage [196,216]. A third, inactive isoform named CEECAM1 or CERCAM does not seem to be directly related to collagen biosynthesis [217]. Contrary to LHs, GLT25D isoforms bear a C-terminal ER-retention sequence [196].

LH1 null mice [184] phenocopy many aspects of the human kEDS and show a decrease in Hyl in many tissues and an increase in the ratio of lysylpyridinoline to hydroxylysylpyridinoline cross-links, indicating a cross-linking defect of type I collagen. The LH1 null mice were flaccid, had gait abnormalities and around 15 % died due to aortic rupture. Global LH2 null mice are embryonically lethal in early development at E10.5 [218]. Another LH2 mutant mouse model with a frameshift mutation identified in human Bruck syndrome 2 patients has been recently generated and these mice survived to later embryonic stage, death occurring between E14.5 and E18.5 and recapitulated many symptoms of Bruck syndrome 2 [219]. LH3 null mice are embryonically lethal around E9.5 and showed a distinct BM phenotype with defective extracellular collagen IV assembly and intracellular collagen IV accumulation [190,191]. This lethal phenotype was found to be specifically caused by the lack of the Glc-T activity of LH3 as mutant mice with a point mutation disrupting only the LH activity of LH3 developed to adulthood and were grossly normal, although some tissue specific defects were observed in the organization of BMs and collagen fibrils, suggesting that the other two LH isoenzymes cannot fully compensate for the loss of LH activity of LH3 [191]. Furthermore, studies on hypomorphic LH3 mice showed that embryonic survival and correct BM assembly were directly correlated with the level of Glc-T activity [191]. Similar to LH3 null mice, the loss of function of GLT25D1 in mice also affects collagen IV [220]. The GLT25D1 null mice are perinatal lethal with a musculoskeletal phenotype with reduced stability and glycosylation and intracellular accumulation of collagen IV. Glycosylation of collagen VI was also found to be affected in the GLT25D1 null mice, but intriguingly, this did not seem to affect its stability or secretion [220].

Taken together, the results obtained from the LH and GLT25D1 mutant mice indicate that Gal-T and Glc-T activities are crucially important for collagen IV synthesis and assembly and the mouse models affecting Gal-T and Glc-T activities display collagen IV and BM phenotypes that are not seen in LH1 or LH2 null mice. GLT25D2 has been shown, by using a null mouse model, to regulate T-cells and to be more susceptible to experimental hepatitis [221], but to our knowledge collagen IV has not been studied in these mice.

The structural characterization of full-length human LH3 showed an elongated dimeric architecture, with each monomer encompassing three domains aligned as beads-on-a-string [222]. The dimer interface, located at the C-terminus of each monomer comprising the domain responsible for LH activity (*i.e*. the LH domain), yields an elongated S-shaped quaternary structure. This domain features the classical DSBH fold common to the 2-oxoglutarate-dependent dioxygenase superfamily [155], but the homo-dimer interface found in LH enzymes is unique, likely with yet uncharacterized specific roles associated with recognition and processing of collagen polypeptides rather than small molecule substrates [222,223]. In addition, and different from C-P4Hs, this domain features an unprecedented Fe^2+^-dependent self-inhibitory mechanism that prevents excess 2-oxoglutarate substrate uncoupling (and associated need for ascorbate to ensure enzymatic turnover), but may also constitute a significant limitation during *in vitro* drug discovery studies [222,224,225]. In this respect, the increasingly available experimental structure data about the different 2-oxoglutarate dioxygenases operating on collagen residues (*i.e*. prolyl and lysyl hydroxylases) can facilitate the identification of structure/function hallmarks of specificity as well as long-range molecular recognition sites required for catalytic processing of the collagen polypeptides.

The N-terminus of LH3, localized at the outmost regions of the elongated quaternary structure, hosts the GT domain, found responsible for the Glc-T activity of multifunctional LHs. This domain is characterized by Rossman fold GT-A type glycosyltransferase architecture, but shares very limited similarity with known glycosyltransferases. The catalytic site features a non-canonical DxxD motif responsible for binding the Mn^2+^ cofactor essential for catalysis and the UDP-α-Glucose donor substrate [222,224]. Site-directed mutagenesis studies, inspired by structural work and by known pathogenic variants, highlighted an extended cooperative network of amino acid residues operating in a concerted manner to facilitate the retaining-type transfer of the glucose moiety on Gal-Hyl, supporting the possibility of extensive long-range interactions, within and beyond the catalytic site boundaries, for the recognition of collagen substrates [224]. Such observations are consistent with the specificity of glycosylation patterns found on different collagen polypeptides, supporting the need for hallmarks of specificity other than promiscuous processing of Gal-Hyl lysines. Additional in-depth biochemical studies are needed to rationalize the mechanisms underlying this substrate specificity.

The LH and GT domains are separated by a second Rossman fold-containing domain, named “accessory” or AC. Despite structurally more similar and sharing very high amino acid sequence conservation with known glycosyltransferases compared to the functional GT domain, the AC domain was not found to have any enzymatic activity *in vitro* [206,222]. Indeed, this domain features most of the essential amino acids required to carry out glycosyltransferase catalytic activity, including a canonical DxD motif for Mn^2+^ binding. However, structural and biochemical studies ruled out the possibility of Mn^2+^ binding at this site. Likewise, site-directed mutagenesis experiments did not support the ability for this domain to bind metal ions. Structural data also highlighted steric hindrance in the region matching the UDP-sugar donor substrate binding site in glycosyltransferases [222]. The strong degree of sequence conservation highlights the possibility that this domain likely contributes to the LH functions through a yet uncharacterized mechanism, possibly by recruiting collagen substrates and/or functioning as a docking module for molecular contacts with binding partners during collagen biosynthesis.

Very recent molecular studies on GLT25D enzymes surprisingly showed remarkable features in these enzymes, partially matching some of the unique features observed in LHs and, in this context, supporting the idea of structure/function hallmarks associated with the entire collagen lysine PTM machinery. Crystal structure and biophysical studies of human full-length GLT25D1 revealed elongated dimers, intertwined by N-terminal flexible segments that stabilize contacts involving the N-terminal inactive GT1 domain, followed by a GT2 domain responsible for the Gal-T catalytic activity [226]. Intriguingly, and similar with what was observed for the GT domain of LHs, the topology and the amino acid network shaping the functional GT2 domain is almost unique of GLT25D enzymes when compared to known glycosyltransferases, highlighting the unique substrate requirements of GLT25D enzymes. Equally surprising is the structural similarity of the non-functional GT1 domain with the AC domain of LHs. Different from LHs, this domain is capable of binding metal ions and donor substrates but cannot perform activation of the UDP-α-Galactose molecule and subsequent transfer to the acceptor substrate due to the absence of essential negatively charged residues common to all inverting-type glycosyltransferases [226].

In summary, both LH and GLT25D enzyme families are characterized by unique features when compared to other hydroxylases/glycosyl-transferases. In particular, both enzymes depend on binding of metal ions for their folding stability other than for catalytic purposes and share dimeric, elongated, multi-domain architectures hosting catalytic portions alternated to accessory elements which do not contribute directly to catalysis. Despite precise functional roles for the accessory elements are yet to be characterized, the high degree of sequence and structural conservation suggests that these domains may likely serve as hubs for long-range interactions with collagen substrate molecules. Surprisingly, these accessory domains share very high structural similarity within the two enzyme families, suggesting evolutionary implications. In particular, they might hinder the existence of a common ancestor displaying all three enzymatic activities, as originally proposed prior to the discovery of GLT25D enzymes [206,215], or possible convergent evolution of distinct enzymatic functions towards collagen substrates through the introduction of a shared module to assist molecular interactions during the subsequent reactions occurring on collagen lysine residues. In this respect, it remains to be seen whether LH and GLT25D enzymes can directly tunnel the product of lysyl hydroxylation from LH into the galactosyltransferase catalytic site of GLT25D, and subsequently back to LH in the GT domain, or if these reactions occur independently through release of the reaction product and independent recognition of the substrates for the next reactions.

Alternatively, these domains may constitute contact sites for protein-protein interactions, enabling the formation of macromolecular complexes. This hypothesis is supported by the absence of ER retention sequences in LHs, highlighting the need for contacts with binding partners to ensure sub-cellular localization during collagen biosynthesis. Intriguingly, LH enzymes were also found in the Golgi apparatus, in the extracellular space, and in collagen IV-containing trans-Golgi vesicles [211,213,214]. This may suggest that, other than being multifunctional enzymes, LHs may exert additional functions in different cellular compartments, possibly linked to molecular interactions with different binding partners. Considering the accumulating evidence associated with aberrant LH activity in the microenvironment of metastatic solid tumors [214,227], advancing our understanding of structure-function relationships of the collagen lysine PTM machineries through the investigation of the interactomes of LH and GLT25D enzymes may also lead to development of innovative cancer therapeutics.

## Other post-translational modifications – disulfide bonds, N-glycosylation and cross-linking

In collagens, most PTMs, as discussed above, occur within the Gly-X-Y collagenous sequence. Besides the lysine and proline modifications, there are several interchain disulfide bonds that can contribute to the collagen structure and stability [228–230]. In addition to these, the central collagenous region of collagen IV harbors a cysteine knot, located approximately 110 nm from the N-terminus, a region known as the CB3 fragment generated by cyanogen bromide digestion [24,228]. Recent study suggests that this cystine knot is evolutionarily conserved across metazoan phyla and plays an important role in stabilizing collagen α1α1α2(IV) protomer at body temperature [228]. In collagen IV, cysteine residues are notably enriched in the N-terminal 7S domain [231,232] and involved in the intermolecular cross-linking of four collagen IV protomers. This process, which leads to the formation of a 7S tetramer (or dodecamer when considering all 12 alpha chains). Although disulfide bond formation can occur spontaneously under an oxidative environment of the ER, however, this reaction is efficiently facilitated by PDI family proteins, comprising approximately 20 members [233,234]. PDI, a tetramer subunit of C-P4Hs, is a prominent disulfide isomerase in this family, However, it remains unknown which PDI family member(s) are responsible for isomerizing each disulfide bond in collagen IV. Further stability can be achieved with non-disulfide cross-linking. It has been suggested that LOXL2 (lysyl oxidase-like-2) contributes to 7S docecamer stability via cross-linking lysine and/or hydroxylysine [235]. N-glycosylation serves as a recognition signal for the ER chaperones calreticulin and calnexin that facilitate proper protein folding in the ER [84,236]. The C-propeptides of fibrillar collagens are N-glycosylated and this modification is known to play a role in their quality control [50]. Notably, the C-propeptide architecture is highly conserved, whereas the N-propeptides of fibrillar collagens exhibit significant diversity [237–239]. While a potential conserved N-glycosylation site is present in the N-propeptides of α1(III), α1(V), and α3(V) in both human and mouse, their domain structures differ. Conversely, in collagen IV, the N-glycosylation site appears within the region of N-terminal 7S domain in most α(IV) chains (α1, α2, α4, α5, and α6), rather than at the C-terminal NC1 domain as seen in fibrillar collagen C-propeptides. In the human α3(IV) chain, however, the N-glycosylation site is located further downstream compared to the conserved site within the 7S domain of other collagen IV α chains, based on UniProt sequence information (UniProt ID: Q01955). The presence of N-glycosylation has been confirmed by NMR and MS analyses for the α1 (IV) and α2(IV) chains and it is proposed to be involved in 7S assembly [136,240]. It is important to note that N-glycosylation rarely occurs within the Gly-X-Y collagenous sequence, as this modification requires an Asn-X-Ser/Thr motif, where X is any amino acid except proline [241]. Indeed, no N-glycosylation sites have been identified in the central collagenous region of collagen IV in humans. Thus, the presence of an N-glycosylation site within the triple-helical sequence, Gly-X-Asn-Gly-Ser/Thr-Y-Gly, in the 7S domain is particularly intriguing. However, the precise role of this modification remains undefined.

As described in the section “NC1 domain assembly for Triple helix propagation”, the C-propeptides of fibrillar collagens undergo N-glycosylation. In contrast, the analogous NC1 domains of collagen IV lack N-glycosylation. Interestingly, there is a unique Hyl-mediated PTM as two NC1 trimers assemble into a stable hexamer in the ECM via sulfilimine cross-links between methionine and lysine residues on opposing NC1 domains [242,243]. Subsequent studies determined that these bonds involve Hyl rather than lysine [243]. Therefore, the lysine needs to be hydroxylated prior to sulfilimine bond formation. We anticipate that this modification occurs in the ER, however, since LHs have been shown to be present in the secretory pathway and extracellular space as described above, it could also occur in the ECM. The enzyme(s) responsible for NC1 lysine hydroxylation remain unknown, and how this lysine PTM contributes to sulfilimine bond formation is likewise uncharted. Further studies are required to elucidate these unique PTMs in collagen IV.

## Advantages of invertebrate model organisms to study collagen IV biosynthesis

Model invertebrate organisms are powerful tools for unravelling biological mechanisms when the target of our biological question is highly conserved in all species. For example, the field of proteostasis is still leveraging these simpler systems, such as bacteria and yeast [244–246]. Is this true for the study in collagen biosynthesis? The answer is yes, particularly for collagen α1α1α2(IV) biosynthesis because it is evolutionarily the most ancient collagen when considering both collagen IV isoforms and the entire collagen superfamily [2,3]. The α1 (IV) and α2(IV) chains are encoded by the *Col4a1* and *vkg* genes in *Drosophila* and the *emb-9* and *let-2* genes in *C. elegans*, respectively. Importantly, these organisms lack fibrillar collagens entirely [1]. While a few additional collagen-related genes exist in *Drosophila* [247], *C. elegans* has over 170 collagen-related genes, most of which encode small collagen-like polypeptides that form the cuticle, the exoskeleton of *C. elegans* [34,247]. Therefore, *Drosophila* collagen α1α1α2(IV) biosynthesis, has a more simple molecular ensemble for collagen modification [1,248]. Similar to collagen genes, the genes encoding the components of the biosynthetic molecular ensemble are largely simplified in *Drosophila*. For instance, LH and GLT25D are each represented by only one isoform (FlyBase IDs FBgn0036147 and FBgn0051915, respectively). Additionally, *Drosophila* has only a single ER-resident FKBP, FKBP14 (FBgn0010470), which resembles human FKBP22. This single gene component allows us to uncover direct evidence of the role in collagen α1α1α2(IV) biosynthesis. Indeed, one good example is examined in *C. elegans*. It has only one LH, LET-268, and, in the absence of LET-268, collagen IV is expressed, however, it is retained within cells, indicating a defect in secretion [249]. Importantly, the LET-268 contains LH and Glc-T activities similar to human LH3 enzyme [250]. Collectively, these organisms are powerful systems for investigating collagen α1α1α2(IV) biosynthesis.

In *Drosophila* P4Hs exhibit greater complexity, with 26 P4H genes cataloged in FlyBase [247]. Genetic studies suggest that prolyl-4-hydroxylase-alpha EFB (FBgn0039776) may function as a *Drosophila* collagen P4H, as prolyl-4-hydroxylase-alpha EFB knockdown significantly affects collagen IV biosynthesis and trafficking [251,252]. On the other hand, a biochemical study showed that recombinant prolyl-4-hydroxylase-alpha MP (FBgn0015713) is capable of hydroxylating collagen peptides [253]. These two P4Hs are promising candidates for collagen IV hydroxylation within the 26-member *Drosophila* P4H superfamily. However, it remains unclear whether one, both or more *Drosophila* P4H enzymes contribute directly or indirectly to collagen α1α1α2(IV) biosynthesis as the puzzle has currently been only partially solved for both of these enzymes.

A remarkable difference between *Drosophila* and humans is that *Drosophila* lacks HSP47. HSP47, a collagen chaperone essential for collagen quality control and navigating collagens to ER exit sites for trafficking in mammals [90,91], is also absent in *Drosophila* [254,255], although HSP47 is present in all vertebrates [256] and ancestral HSP47 is identified in Japanese lamprey (Lethenteron japonicum) [254]. Interestingly, *Drosophila* possesses TANGO1 [102], that directs HSP47/collagen complexes to transport vesicles at ER exit sites in mammals [108]. While TANGO1 plays an essential role in ECM protein trafficking in *Drosophila* [106,252,255], the mechanism by which Drosophila collagen α1α1α2(IV) is secreted in the absence of HSP47 is not fully understood. A potential explanation is that the SH3 domain of *Drosophila* TANGO1 may bind directly to collagen IV [257]. However, it remains unclear whether this binding occurs to intact triple-helical collagen IV. This is because the collagen IV used in the studies was isolated through pepsin digestion and prepared at 37 °C for binding assays [257,258], which could potentially alter its native structure.

*Drosophila* also lacks P3H, which provides significant proline hydroxylation necessary for mammalian collagen α1α1α2(IV) functionality [22,163,165]. It is intriguing to note that P3H2 is evolutionarily oldest P3H isoform [259] and is involved in collagen α1α1α2(IV) biosynthesis, as described above. *C. elegans* also lacks P3H, according to in-silico characterization [260]. Further characterization is required to identify the evolutionary origin of the P3H enzyme. LH3 and P4HA1 are considered the evolutionary ancestors of the LH and C-P4H isoforms [259,261], which is convincing given that these enzymes are critical for collagen IV and the BM formation [44,135,156,190].

Collectively, we propose that the collagen α1α1α2(IV) biosynthetic molecular ensemble in *Drosophila* represents a simpler, yet efficient mechanism for collagen biosynthesis that may reflect a minimum from an evolutionary perspective.

## Pathology associated with collagen IV and PTMs

Collagen IV networks are affected in a range of human genetic disorders leading to BM, tissue and organ dysfunction (Table 2). Gould syndrome is a rare genetic disorder caused by heterozygous variants in *COL4A1* and C*OL4A2* and impacting the α1 and α2 chains of collagen IV. These variants affect the secretion, assembly and interactions of the α1α1α2(IV) network that are vital for BM function across multiple tissues [262]. Clinical phenotypes range from porencephaly to muscular dystrophy and ocular abnormalities, highlighting the role of the α1α1α2 (IV) network in neural and muscular tissue development and maintenance [263]. The α1α1α2(IV) network is ubiquitous and this likely explains the range of phenotypes across many tissues, the dominant effect of heterozygous variants and the lethality of homozygous variants. The α3α4α5(IV) and α5α5α6(IV) networks have tissue restricted expression and their specific roles are uncertain, although the emergence of the α3α4α5(IV) network during evolution coincided with kidney filtration [264]. Alport syndrome is another rare genetic disorder that predominantly affects the kidneys, eyes, and ears, and it is caused by variants in the Alport genes: *COL4A3, COL4A4*, and *COL4A5*, which are necessary for the tissue restricted α3α4α5(IV) network. Variants in the Alport genes do impair the long-term structural integrity of selected BMs, leading to progressive kidney disease but also hearing loss, and defects in retinal and lens BMs in the eye [265].

Alongside these genetic disorders that affect the collagen IV sequence directly, there are also overlapping phenotypes associated with the enzymes that modify collagen IV. The C-P4H and P3H modifications of proline are examples. Biallelic variants in *P4HA1* result in an early-onset disorder characterised by joint contractures, hypotonia, osteopenia and high myopia [266]. Analyses of muscle biopsies from the proband reported in this study showed reduced collagen IV immuno-reactivity in the muscle BM. Interestingly the muscle phenotype in the proband was severe at birth and improved over time. The authors hypothesised that different requirements of the affected *P4HA1* splice isoforms during development could account for the clinical improvement. A penetrant heterozygous phenotype in this study was high myopia or short sightedness and this eye phenotype is also observed with heterozygous *P4HA2* variants [267]. The high myopia has been attributed to elongation of the eye as a consequence of collagen structural abnormalities [268]. Whilst high myopia has not been reported in Gould syndrome, the phenotypes observed with *P4HA1* and *P4HA2* variants have some overlap in the tissues affected in Gould syndrome, suggesting that the phenotypes may be a consequence of effects on collagen IV, which is supported by C-P4H mutant mouse studies described above.

In contrast, variants in *P3H2* have been associated with defects in the glomerular BM in the kidney, thereby overlapping with the phenotypic spectrum of Alport syndrome. Variants in *P3H2* can lead to a thinner glomerular BM, causing microscopic haematuria and albuminuria over time and this is consistent with an effect on the collagen IV network in the glomerular BM [165]. The mechanism of P3H2 pathogenicity was investigated in P3H2 null mice and the modification of the collagen IV network catalyzed by P3H2 was found to enable important interactions. There was embryonic lethality due to loss of interactions between non-prolyl-3-hydroxylated type IV collagen and maternal GPVI, leading to thrombosis and furthermore the double knockout of *P3H2* and *GPVI* rescued this phenotype [163]. A *P3H2* variant that leads to complete inactivation of the enzyme has also been shown to cause high myopia [180]. Therefore, the range of human phenotypes associated with proline modification, either 4Hyp or 3Hyp, of collagens is wide and there is an emerging association with phenotypes that result from effects on collagen IV networks.

Since lysine modification is also vital for the function of many collagens, genetic defects in the genes that encode the lysine modifying enzymes also have phenotypic overlap with the relevant collagen and this leads to a range of human phenotypes from muscle, skin and skeletal disorders to cancer. As described earlier, variants in *PLOD1* are associated with EDS, a large group of multisystem disorders, in which variants in collagen V genes cause the classical EDS type [269]. The *PLOD1*-related EDS type, kEDS, is characterised by hypotonia, generalised joint hypermobility, early-onset kyphoscoliosis, skin fragility, and ocular abnormalities [187] and this demonstrates a phenotypic overlap between the predominant collagen and the lysine modifying enzymes.

*PLOD3* variants have been reported to result in a complex connective tissue disorder, the phenotype overlapping with many collagen disorders [270–273]. Interestingly, *PLOD3* has a more direct association with a BM phenotype as it has been shown to be associated also with epidermolysis bullosa (EB), a genetic skin fragility disorder. Recessive dystrophic EB (RDEB) is typically associated with variants in *COL7A1* which lead to blistering beneath the BM due to disruptions in anchoring fibrils. A homozygous missense variant in *PLOD3* was identified in an individual with RDEB but no associated variant in *COL7A1* [273]. The individual reported in this study had significantly reduced LH3 levels and there was a marked decrease in type VII collagen expression and its glycosylated derivatives. LH3 deficiency has also been shown to impair the secretion of collagen IV but not collagens I or III [44,192]. In this study amino acid analysis found that LH3 is a selective LH for collagen IV and rare variants in *PLOD3* that are predicted to be pathogenic were associated with intracranial haemorrhage and therefore overlapping with the phenotypic spectrum of Gould syndrome [274].

Variants in the collagen galactosyltransferase gene *COLGALT1* are also associated with musculoskeletal and cerebrovascular phenotypes, exhibiting further phenotypic overlap with Gould syndrome [275,276]. As research progresses and more of the ~20 genes required for collagen IV synthesis and assembly are incorporated into genomic studies, additional human phenotypes related to collagen IV modifications will emerge. Already, with our knowledge of collagen IV biosynthesis it is possible to apply reverse phenotyping [277] to identify individuals with variants in the biosynthetic genes and to determine whether there are associated phenotypes that overlap with collagen IV related disorders.

Abnormal accumulation of collagen and replacement of functional organ tissue with connective tissue are hallmarks of fibrosis, ultimately leading to organ failure. Although fibrillar collagens are typically implicated in fibrosis, BM remodelling is also involved in, for example, pulmonary, kidney and corneal fibrosis and liver cirrhosis and circulating levels of collagen IV or its fragments are increased in individuals with idiopathic pulmonary fibrosis and hepatitis [278–281]. Furthermore, upregulation of the expression of genes encoding collagen biosynthesis enzymes is observed in fibrotic conditions, in particular those for C-P4H required in the synthesis of all collagen types, and LH2 and the extracellular cross-linking enzyme lysyl oxidase that contribute to increased fibrillar collagen cross-linking and ECM stiffness [202,282,283]. Collagens and ECM remodelling have important roles also in tumour growth and metastasis. Overexpression of collagen hydroxylases and their aberrant activity is observed in several cancer types and are often regarded as markers of bad prognosis and suggesting opportunities for drug targeting [284–289]. Although the range of the affected target collagens is yet less well understood, in addition to alterations in the organization of fibrillar collagens in tumors, aberrantly post-translationally modified circulating collagen IV and its fragments in metastatic cancers has been reported [290–292]. These observations highlight the need for in-depth characterization of the collagen IV PTM machinery and further studies are required to understand their roles in fibrosis and cancer.

With the increasing gene to phenotype associations described for collagen IV and the ensemble of modifying proteins, it is important to build strong genetic and experimental evidence of causality. Since collagen genes are highly conserved, the function of modifying proteins can be studied in a range of vertebrate animal systems. In zebrafish *p3h1* deficiency leads to an osteogenesis imperfecta phenotype [293] and the effects of P3H on collagen IV could also be investigated in this system. In mouse models, as described above, genetic total or partial silencing of *P3h1, P3h2, P3h3, P4ha1, P4ha2, Plod1, Plod2* or *Plod3* results in phenotypical changes that mirror human pathologies related to fibrillar collagens and collagen IV, including abnormal skeletal development, defects in BMs or skin fragility. Similarly, defects in the *Colgalt1* gene in animal models replicate the musculoskeletal and cerebrovascular manifestations seen in humans with *COL4A1* and *COL4A2* variants, highlighting the importance of collagen glycosylation in maintaining vascular integrity and muscle function [220]. The knowledge we acquire from animal models will enable greater understanding of collagen IV biosynthesis and the associated disease mechanisms and in turn this will guide the development of therapeutic strategies.

Prospects for treatment related to collagen IV related disorders are promising. Gene therapies present potential strategies to target specific variants affecting the collagen IV genes, aiming to restore normal gene function by correcting or compensating for the defective genes but there are challenges with the size of the *COL4A* genes [294]. Additionally, chaperone therapies may help stabilize misfolded collagen proteins, preventing their degradation and preserving their function, potentially mitigating phenotypes associated with protein misfolding [295]. The development of specific therapies that correct defects in PTMs, such as hydroxylation and glycosylation could also be a future therapeutic strategy.

The exploration of collagen IV biosynthesis and the associated pathologies through human studies and diverse animal and cell-based models is enriching our understanding of the biological functions of collagen IV. This foundational knowledge is crucial for unravelling the molecular mechanisms behind associated diseases and developing innovative therapeutic strategies. As our comprehension of collagen IV and the relevant biosynthetic genes continues to evolve, it will be possible to have more refined disease classification and through patient registries to identify individuals who may benefit from an array of emerging gene- and protein-based therapeutics.

## Summary

In this review we have illustrated that the collagen IV molecular biosynthesis ensemble is an intricate process that interplays a complex array of assembly, folding, and post-translational modifications to achieve its proper structure and function within BM. The specialized enzymatic machinery governing these modifications not only distinguishes collagen IV from other collagen types but also underlies its critical role in tissue homeostasis and disease. We summarize our discussions above and present a table that visualizes the function-based components of the collagen IV molecular biosynthesis ensemble, along with the magnitude of their distribution compared to the collagen I molecular biosynthesis ensemble (Table 1). A deeper understanding of these unique molecular pathways promises to open new avenues for therapeutic intervention in collagen-related disorders.

## Outstanding questions

1. How do collagen substrates bind to their modifying enzymes? Are there long-range interactions enabling molecular recognition?
2. Do any trafficing regulators exist for P4Hs, P3Hs, and LHs to access X-Y-Gly triplets? Is the accessibility of these enzymes to the triplets competitive or cooperative?
3. Why do the levels of 3Hyp, Hyl, Gal-Hyl, and Glc-Gal-Hyl differ among collagens and also in the same collagen type in different tissues? What modulates these levels? Are there any rules governing these differences?
4. How do the PSB and CAT domains in C-P4H cooperate?
5. What are the hallmarks of LH/GLT25D cooperativity in processing lysine substrates ultimately leading to Glc-Gal-Hyl?
6. Are there roles for the non-functional evolutionarily related AC domain in LH and GT1 domain in GLT25D?
7. Why is dysfunction in collagen modifying enzymes implicated in pathology? Specifically, does the absence of PTMs, such as sugars or hydroxyl groups, directly contribute to disease? Or is the impact secondary, manifesting through mechanisms such as damage to the structural properties of collagen IV, prevention of interactions between collagen IV and ECM proteins, impairment of collagen IV biosynthesis, or disruption of the molecular biosynthetic ensemble of collagen IV?

## Figures and Tables

**Table 1:** Quick reference chart of “function-based” molecular contributions for collagen IV and collagen I molecular ensembles.

Sub-category	Protein name (*Gene name*)		Molecular ensemble	
			Col IV	Col 1
Chaperone	HSP47 (*SERPINH1*)		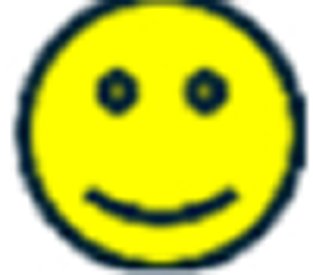	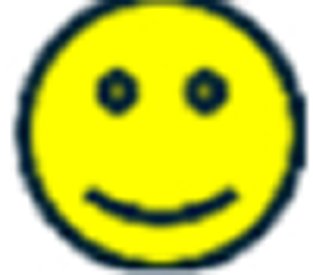
Prolyl 4-hydroxylase (P4H)	P4HA1 (*P4HA1*)		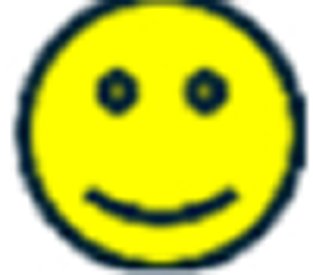	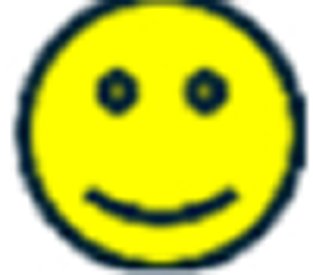
	P4HA2 (*P4HA2*)		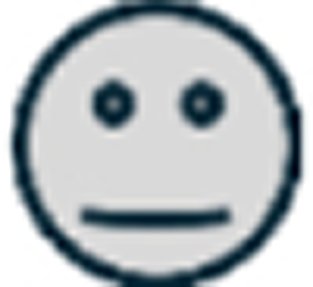	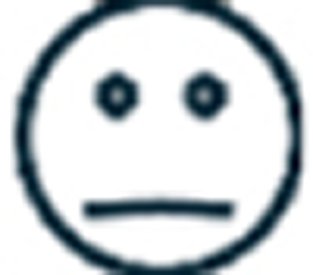
	P4HA3 (*P4HA3*)		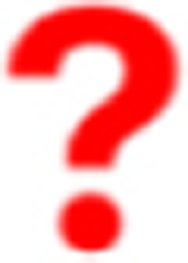	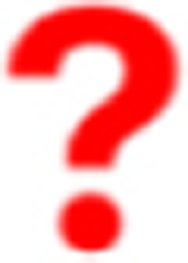
Prolyl 3-hydroxylase (P3H)	P3H1 (*P3H1* or *LEPRE1*)		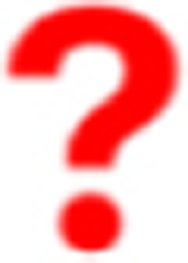	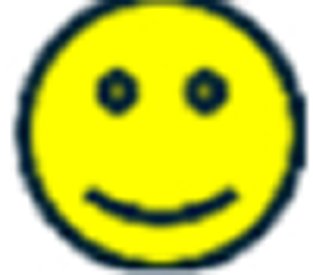
	P3H2 (*P3H2* or *LEPREL1*)		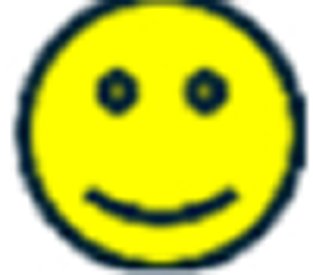	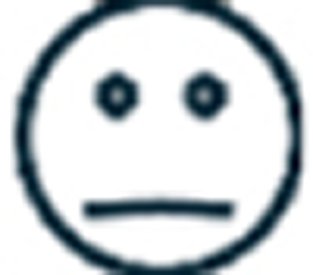
	P3H3 (*P3H3* or *LEPREL2*)		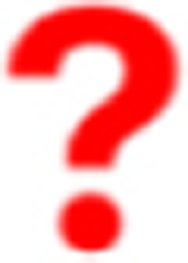	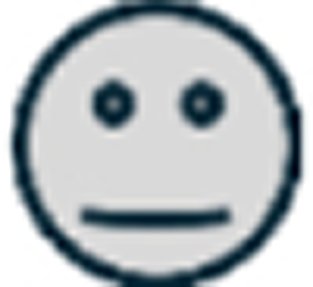
Lysyl hydroxylase (LH)	LH1 (*PLOD1*)		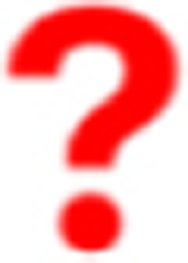	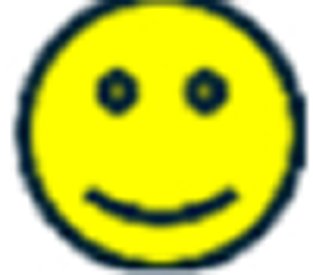
	LH2 (*PLOD2*)		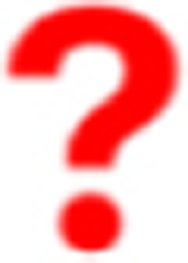	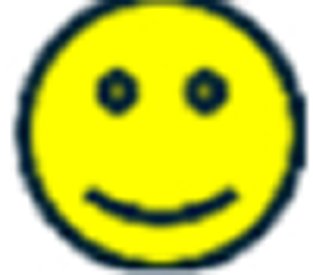
	LH3 (*PLOD3*)		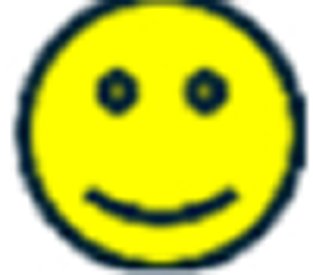	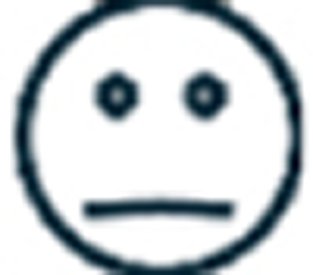
Galactosyltransferase	GLT25D1 (*COLGALT1*)		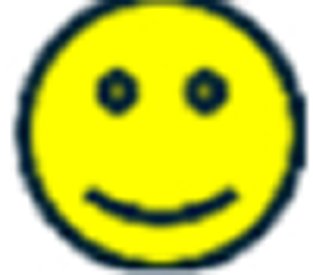	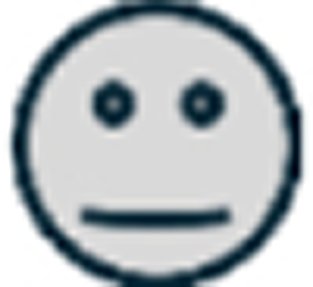
	GLT25D2 (*COLGALT2*)		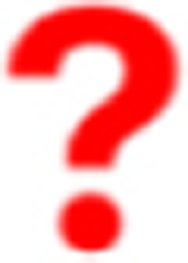	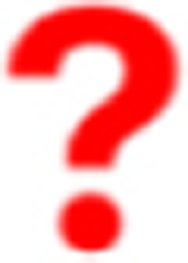
Glucosyltransferase	LH3 (*PLOD3*)		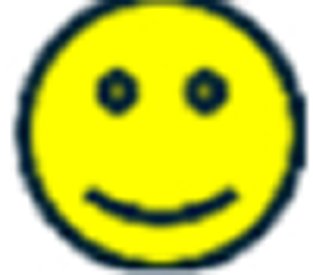	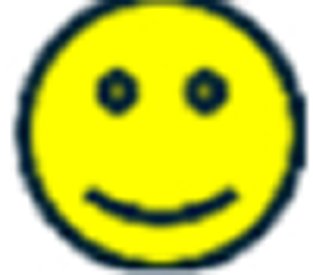
Prolyl isomerase	FKBP65 (*FKBP10*)	Prolyl isomerase	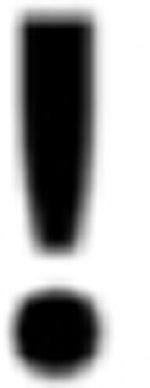	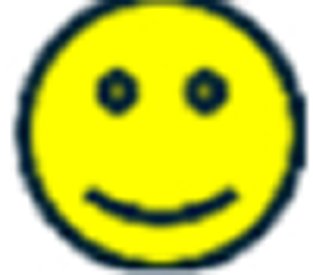
		Chaperone	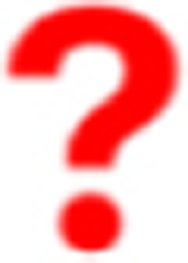	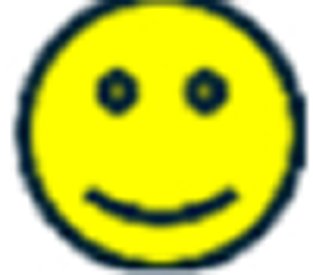
	FKBP22 (*FKBP14*)	Prolyl isomerase	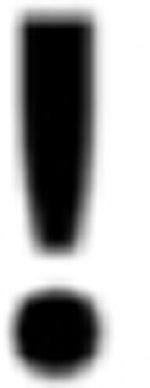	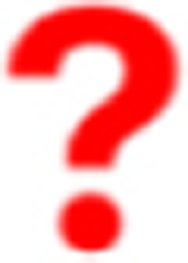
		Chaperone	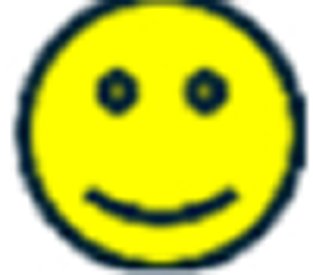	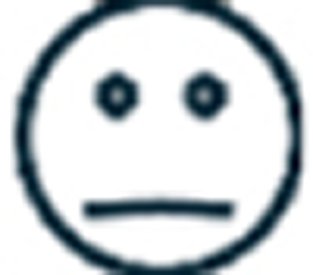
	CYPB (*PPIB*)		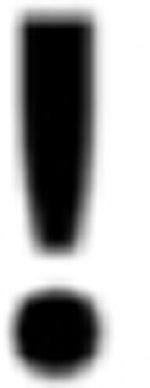	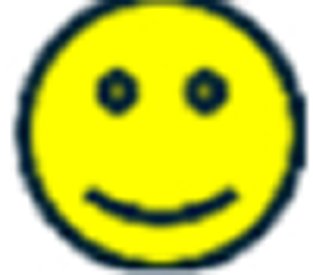
Other	CRTAP (*CRTAP* or *CASP*)		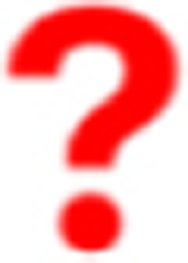	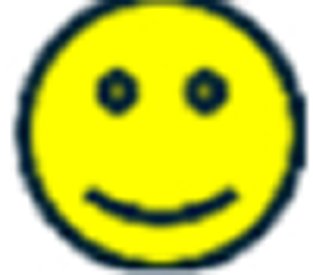
	SC65 (P3H4 or *LEPREL4*)		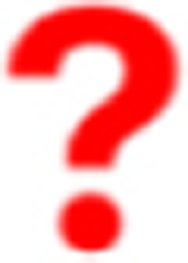	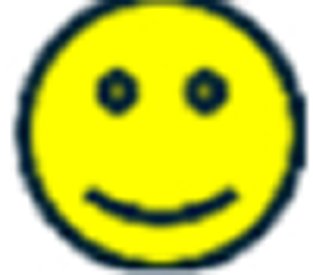

Note: Each symbol indicates a function-based contribution of the molecular biosynthesis ensemble in collagen IV (Col IV) and collagen I (Col I) − 
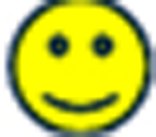
: high,
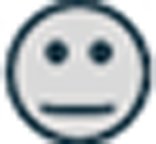
: middle,
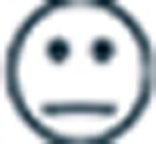
: low,
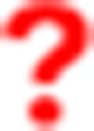
: unknown and ! : predicted.

**Table 2:** Human disease phenotypes associated with collagen IV and enzymes that modify intracellular collagen IV.

Sub-category	Protein name	Gene name	Human phenotypes	Disease name
Collagen IV	Collagen alpha-1 (IV)	*COL4A1*	1. Brain small vessel disease 1 with or without occular abnormalities. 2. Hereditary angiopathy with nephropathy, aneurysms and muscle cramps. 3. Susceptibility to intracerebral haemorrhage. 4. Tortuosity of retinal arteries. 5. Autosomal dominant pontine microangiopathy and leucoencephalopathy	Gould syndrome
	Collagen alpha-2 (IV)	*COL4A2*	1. Brain small vessel disease 2. 2. Susceptibility to intracerebral haemorrhage	Gould syndrome
	Collagen alpha 3 (IV)	*COL4A3*	1. Kidney disease. 2. Sensorineural hearing loss. 3. Ocular abnormalities	Autosomal Alport syndrome
	Collagen alpha-4 (IV)	*COL4A4*	1. Kidney disease. 2. Sensorineural hearing loss. 3. Ocular abnormalities	Autosomal Alport syndrome
	Collagen alpha5 (IV)	*COL4A5*	1. Kidney disease. 2. Sensorineural hearing loss. 3. Ocular abnormalities	X-linked Alport syndrome
	Collagen alpha-6 (IV)	*COL4A6*	1. Hearing loss 2. Leiomyomatosis (when there is a contiguous deletion with COL4A5)	X-linked hearing loss
Chaperone	HSP47	*SERPINH1*	1. Preterm premature rupture of membranes. 2. Osteogenesis imperfecta, Type X	Osteogenesis imperfecta
Prolyl 4-hydroxylase	P4HA1	*P4H1*	1. Joint contractures, 2. hypotonia, 3. osteopenia 4. high myopia	None
	P4HA2	*P4H2*	1. High myopia	None
	P4HA3	*P4H3*	Not described	None
Prolyl 3-hydroxylase	P3H1	*P3H1 or LEPRE1*	1. Osteogenesis imperfecta, type VIII	Osteogenesis imperfecta
	P3H2	*P3H2 or LEPREL1*	1. Myopia, high, with cataract and vitreoretinal degeneration, 2. Glomerular basement membrane defects	None
	P3H3	*P3H3* *or* *LEPREL2*	Not described	None
Lysyl hydroxylase	LH1	*PLOD1*	Ehlers-Danlos syndrome, kyphoscoliotic type 1	Ehlers-Danlos syndrome
	LH2	*PLOD2*	Osteogenesis phenotypic spectrum	Bruck syndrome 2
	LH3	*PLOD3*	1. Bone abnormalities, 2. Cataract, 3. Arterial rupture, 4. Deafness. 5. Recessive dystrophic epidermolysis bullosa	BCARD syndrome
Galactosyl- transferase	GLT25D1	*COLGALT1*	1. Musculoskeletal defects, 2. Cerebrovascular defects	None
	GLT25D2	*COLGALT2*	Not described	None
Glucosyl-transferase	LH3	*PLOD3*	1. Bone abnormalities, 2. Cataract, 3. Arterial rupture, 4. Deafness. 5. Recessive dystrophic epidermolysis bullosa	BCARD syndrome
Prolyl isomerase	FKBP65	*FKBP10*	Osteogenesis phenotypic spectrum	Bruck syndrome 1
	FKBP22	*FKBP14*	Ehlers-Danlos syndrome, kyphoscoliotic type 2	Ehlers-Danlos syndrome
	CYPB	*PPIB*	Osteogenesis imperfecta, type IX	Osteogenesis imperfecta
Other	CRTAP (CRTAP or CASP)	*CRTAP or CASP*	Osteogenesis imperfecta, type VII	Osteogenesis imperfecta
	SC65 (P3H4 or LEPREL4)	*P3H4 or LEPREL4*	Not described	None

## Data Availability

No data was used for the research described in the article.
